# Next-generation sequencing as an approach to dairy starter selection

**DOI:** 10.1007/s13594-015-0227-4

**Published:** 2015-04-24

**Authors:** Philip Kelleher, James Murphy, Jennifer Mahony, Douwe van Sinderen

**Affiliations:** 1 School of Microbiology, University College Cork, Cork, Ireland; 2 Alimentary Pharmabiotic Centre, Biosciences Institute, University College Cork, Cork, Ireland

**Keywords:** LAB, *Lactococcus*, *Streptococcus*, Dairy fermentation, Phage, Genomics

## Abstract

Lactococcal and streptococcal starter strains are crucial ingredients to manufacture fermented dairy products. As commercial starter culture suppliers and dairy producers attempt to overcome issues of phage sensitivity and develop new product ranges, there is an ever increasing need to improve technologies for the rational selection of novel starter culture blends. Whole genome sequencing, spurred on by recent advances in next-generation sequencing platforms, is a promising approach to facilitate rapid identification and selection of such strains based on gene-trait matching. This review provides a comprehensive overview of the available methodologies to analyse the technological potential of candidate starter strains and highlights recent advances in the area of dairy starter genomics.

## Introduction

The first origins of fermented dairy products can be traced back over 7000 years to the pottery sieves used by some of the first farmers in Europe (Curry 2013). While the dairy industry has evolved since then, the same two basic ingredients remain its cornerstone: milk and lactic acid bacteria (LAB). LAB produce lactic acid from the degradation of hexose sugars and have a long history of safe use in food fermentations, and consequently, many LAB have been granted the so-called generally regarded as safe (“GRAS”) status (Wegmann et al. 2007). LAB encompass an array of bacterial genera including *Abiotrophia*, *Aerococcus*, *Carnobacterium*, *Enterococcus*, *Lactobacillus*, *Lactococcus*, *Leuconostoc*, *Oenococcus*, *Pediococcus*, *Streptococcus*, *Tetragenococcus*, *Vagococcus* and *Weissella* (Makarova et al. 2006). The most frequently employed starter cultures in dairy fermentations are strains of *Lactococcus lactis* and *Streptococcus thermophilus*, while strains of *Lactobacillus* spp. are primarily employed as adjunct cultures for flavour development (Leroy and De Vuyst 2004).

The earliest dairy fermentations occurred through the activity of the autochthonous organisms present in milk, thus causing spontaneous fermentation. The success of such natural fermentations is variable and depends on the particular natural blend of microorganisms present in each batch. The first attempt to control these fermentations was borne out by the “back-slopping” technique which involves inoculating milk with a small amount of a successful fermentate thus seeding the milk with favourable bacteria. This practice is still commonly applied to produce certain artisanal cheese products, and in such cases, the starter culture mixes are termed undefined starters. In industrial dairy fermentations, starter cultures may be defined or undefined. Defined starter cultures are those which consist of a specific number of known strains of bacteria which had been isolated from undefined starter cultures, while undefined are those of which the constituent strains are unknown (Smid et al. 2014). In Italian artisanal cheeses where back-slopping is employed, starter cultures are said to be undefined starters, while most cheddar-type cheeses are produced with a defined set that consist of two to four strains. The application of defined starter culture blends and rotations aims to ensure consistency of the fermentation process and particularly the quality of the final product in terms of flavour, appearance, aroma and safety.

This review will focus on *L. lactis* and *S. thermophilus* as the dominant starter cultures employed by the dairy industry (Beresford et al. 2001). In particular, it will focus on the methods used to characterise these strains and on the role of next-generation sequencing (NGS) technologies in facilitating rational starter culture selections and rotations.

*L. lactis* is a Gram-positive, catalase-negative, non-motile and coccoid bacterium (Schleifer and Kilpper-Bälz 1987). Genetically, a typical *L. lactis* chromosome ranges in size from ~2.2 to 2.5 Mb and is often accompanied by a rich plasmid complement (Ainsworth et al. 2014c). *L. lactis* species can be further defined as subsp. *cremoris*, subsp. *lactis* or subsp. *lactis* biovar. diacetylactis, the latter capable of metabolising citrate. Both citrate metabolism and lactose utilisation are plasmid-encoded traits in *L. lactis*. Notably in dairy isolates of *L. lactis*, genome decay and redundancy have been widely reported (Makarova et al. 2006; Goh et al. 2011; Ainsworth et al. 2013), along with the presence of prophages and a host of transposable elements (Chopin et al. 2001).

*S. thermophilus* strains are Gram-positive, catalase-negative and non-motile cocci (Schleifer and Kilpper-Bälz 1987), which contain chromosomes that are comparably smaller than their *L. lactis* counterparts (1.8 Mb compared to 2.5 Mb), and normally, about 59% of strains carry one or two small plasmids (<10 kb) (Turgeon and Moineau 2001).

## Traditional methods for screening/selection of starter strains

There is a wide variety of microbiological techniques that have been used in the dairy industry to screen and define starter cultures. Traditionally, the majority of these methods have, unsurprisingly, placed significant emphasis on the technological phenotypes of the cultures and include growth performance or activity testing, phage robustness, flavour testing and matrix formation analysis.

### Strain differentiation

For dairy lactococcal strains, an early characterisation step is to assign a subspecies, *lactis* or *cremoris*. The major phenotypic differentiator between *L. lactis* ssp. *lactis* and *cremoris* is their adaptive stress response. There are a number of protocols to differentiate between the two sub-species based on temperature, salt tolerance, pH tolerance, maltose fermentation and arginine deamination. One such protocol, based on arginine deamination, is the arginine broth assay described by Harrigan (1998) in which the sub-species identity is determined based on a pH-dependent colour change. *L. lactis* ssp. *lactis* strains are typically capable of metabolising arginine, a process that causes the release of ammonia and concomitant increase in pH, thereby producing red coloured colonies on arginine broth plates (due to a pH indicator). In contrast, *L. lactis* ssp. *cremoris* strains, which normally do not metabolise arginine, produce yellow coloured colonies on this medium because, compared to *L. lactis* ssp. *lactis* strains, their growth results in a lower pH (Villani et al. 2001; Murphy et al. 2013). A number of studies have focused on the response of strains of either sub-species to salt, temperature and pH, where *L. lactis* ssp. *lactis* strains can typically tolerate 4% salt, pH 9.2 and temperatures of up to 40 °C, while *L. lactis* ssp. *cremoris* growth is typically inhibited by these challenging conditions (Schleifer et al. 1985).

### Performance testing

An important aspect of selecting strains for use as starter cultures is performance testing, in which the growth rate, acid production and responses to temperature and salt are assessed. These criteria are an early indicator of how the strains will behave in a fermentation process. The “Pearce activity test” is commonly used in the dairy industry as an indicator for growth and temperature-induced autolysis of starter strains (Feirtag and McKay 1987). The test replicates the temperature cycles of the relevant cheese production process, while monitoring cell counts and pH. The change in pH indicates the acidification activity of the strain, while the point of temperature-induced autolysis is determined via a decrease in cell count after a temperature shift. This test can be employed to assess the level of autolysis, as was done for two dairy starter strains, *L. lactis* subsp. *cremoris* HP and *L. lactis* subsp. *cremoris* AM2, and to determine the resulting proteolytic enzymes released during such autolysis (Wilkinson et al. 1994). Furthermore, the intracellular enzyme lactate dehydrogenase (LDH) can be used as an indicator of autolysis in dairy starter strains (Horvath and Barrangou 2010; Karginov and Hannon 2010), using a method proposed by Wittenberger and Angelo (1970), where LDH is measured by a decrease in absorbance at 340 nm as a result of pyruvate-dependent oxidation of NADH.

### Flavour capabilities

The contribution of lactococcal and streptococcal starter strains to cheese flavour development is predominantly through the metabolism of lactose, lactate, citrate, lipids, proteins and free amino acids (McSweeney 2004). A number of approaches have been employed to assess the contribution of particular starter strains to the relevant pathways, ranging from simple culture techniques to biochemical profiling of key enzymes, and quantitative analysis of sample products using various experimental approaches and/or sensory panels.

Proteolysis is the most complex and possibly the most important of these processes in terms of primary flavour development in cheese. Additionally, it is responsible for the liberation of peptides and (then) amino acids, thereby supplying substrates for various secondary pathways of amino acid catabolism (McSweeney 2004). To assess the proteolytic capabilities of starter cultures, a frequently used simple, direct test is to determine the degree of hydrolysis (DH) of peptide bonds, which provides a measure for amino acid availability and corresponding catabolism in milk (Mosher et al. 2013). Three commonly used methods for the determination of the degree of hydrolysis involve the application of trinitrobenzenesulphonic acid (TNBS) and o-phthaldialdehyde (OPA), which both react with the amino groups released by the hydrolysis of peptides, and pH stat, a titration method where the protons released during peptide bond hydrolysis are used to quantify the DH (Quail et al. 2012). These methods give an overall evaluation of the proteolytic capability of the strains. An alternative method to determine the activity of specific intracellular peptidases was developed by Kato et al. (1978). The method utilises substrates labelled with 7-amino-4-methyl coumarin (AMC), which when cleaved fluoresces at excitation and emission wavelengths of 370 and 440 nm, respectively. The intensity of the fluorescence is used to determine the peptidase concentration. This method has been employed in a number of studies involving lactococci and lactic streptococci with particular emphasis on X-propyl and post-proline dipeptidyl aminopeptidase activity (Mills et al. 2010; Millen et al. 2012; Schloss et al. 2015).

LAB produce aroma compounds through amino acid catabolism, which contribute to cheese flavour, and the activity of glutamate dehydrogenase (GDH) in these strains is closely related to their ability to produce such compounds (Tanous et al. 2002). GDH produces α-ketoglutarate, a compound required for amino acid transaminations by LAB, which is an important step for the synthesis of various amino acids (McSweeney 2004). GDH activity in dairy LAB is usually assessed using a colourimetric assay which measures the glutamate-dependent reduction of NAD^+^ and NADP^+^ in a coupled reaction with diaphorase (Kieronczyk et al. 2003). Amino acid transferases catalyse the transamination of amino acids to α-ketoacids using α-ketoglutarate as an α-ketoacid acceptor (Tanous et al. 2002). These analyses applied in-parallel can provide a detailed biochemical profile of the flavour capabilities of particular strains, though they are limited in providing information that explain the (genetic and/or biochemical) reasons for strain-specific flavour properties.

### Matrix formation

Exopolysaccharides (EPS) produced by LAB are polysaccharides that are deposited outside the cell wall. EPS may remain attached to the cell as capsular EPS or secreted in an unattached form (Hassan et al. 2003). Dairy strains are generally described as “ropy” or “non-ropy”, a term which describes the threads drawn with a needle from the surface of the colonies or fermented liquid (Hassan 2008). The EPS produced by some dairy LAB can impact on the protein matrix of fermented dairy products by affecting the casein gel structure and acting as a filler (Hassan et al. 1995). A common assay for the differentiation of ropy and non-ropy colonies utilises ruthenium red stain in milk agar plates. Ruthenium red stains the cell wall, thereby producing red colonies for non-ropy, non-EPS producing cells, yet is less well capable of staining cell walls of ropy, EPS producers, which thus remain predominantly white (Borucki et al. 2003).

### Phage robustness

While the technological attributes of dairy starter cultures are essential to achieve the desired flavours and characteristics in the final product, the phage robustness of these strains is also an important consideration. Since the discovery of lactococcal (bacterio)phages (i.e. viruses that infect bacterial cells) by Whitehead and Cox in 1935, phage infection has been recognized as the main cause of fermentation problems within the dairy industry with concomitant economic problems (Whitehead and Cox 1935). Selecting the correct starter culture traditionally involved assessing their susceptibility to phage infection, but with the advent of modern sequencing technologies, strain suppliers can now also screen strains to determine the presence of prophages as well as the arsenal of plasmid- and/or chromosomally encoded phage resistance mechanisms (Allison and Klaenhammer 1998; Ainsworth et al. 2014a, b).

The phage life cycle may take one of two possible routes, i.e. the lytic or the temperate/lysogenic life cycle, depending on the phage and the environmental circumstances. Ultimately, all phage species must enter the lytic cycle to harness the host machinery, replicate and release progeny phages by lysing the host cell. However, conditions may not favour lytic replication, and as a result, some phages may engage in a lysogenic life cycle by incorporating their genomes within that of the host, allowing phage replication in situ with that of the host’s genome. This process allows the phage to replicate ‘silently’ each time the bacterium undergoes cell division by binary fission. Under certain conditions, the lysogenic phage will excise from the host genome and enter the lytic cycle. When selecting appropriate starter cultures for the production of various dairy products, it is important to determine if a particular strain harbours any prophages as these pose the risk of becoming active during the fermentation process leading partial or complete culture lysis. The presence of prophages can be determined by phage induction assays whereby the bacterium is placed under particular stressful conditions (chemical treatment or exposure to UV-light) in order to stimulate excision of the integrated phage which will subsequently turn on its lytic life cycle and lyse the host cell (Chopin et al. 2001).

### Bacteriocin production

Bacteriocins are a diverse group of ribosomally synthesized peptides, produced by some bacteria and archaea, which convey a bactericidal or bacteriostatic effect on other bacteria when secreted (Dobson et al. 2012). Bacteriocin production is a double-edged sword, and consideration should be given in selecting starter cultures, as producing strains may inhibit other desirable strains in mixed starter cultures or adjunct cultures added later in the fermentation process. However, they also offer the benefit of inhibiting the growth of spoilage bacteria in food products. Traditionally, a range of culture-based methods have been used in screening for bacteriocin producers, most commonly based on the principles of diffusion in agar plates and cell-free supernatants (Kékessy and Piguet 1970; Barefoot and Klaenhammer 1983; Yang et al. 1992).

## Modern methods for screening/selection of starter strains

Culture-based techniques are an excellent foundation for the study of LAB strains but are both labour- and time-intensive. While these methods have limitations, they can still be applied on selected strains, yet are impractical for screening large strain collections. Current approaches to identify, classify and characterise industrial starter cultures for application in the dairy industry is increasingly reliant on molecular techniques because phenotypic traits alone are not sufficiently discriminative since some strains of *L. lactis* ssp. *cremoris* display the characteristics of *L. lactis* ssp. *lactis* (Urbach et al. 1997; Tailliez et al. 1998). Molecular tools possess several advantages over classical approaches including a shorter assay period, higher throughput and a greater ability to discriminate at the species level. Up until recently, the gold standard of molecular typing of bacterial isolates was pulsed field gel electrophoresis (PFGE). Other methodologies rely on a polymerase chain reaction (PCR) which employs oligonucleotide primers to amplify DNA fragments. Such techniques include PCR-restriction fragment length polymorphism (PCR-RFLP), repetitive sequence-bases PCR (REP-PCR), PCR-ribotyping, amplified fragment length polymorphism (AFLP), random amplified polymorphic DNA (RAPD) PCR and multilocus sequence typing (MLST). As such methods have been discussed extensively, we will only provide a brief overview of the advantages and/or disadvantages of each technique in the context of selecting industrially important dairy starter cultures.

### Restriction endonuclease-based methods

The use of PFGE allows for the separation of large DNA fragments, produced by one or more suitable restriction endonucleases (REases), employing alternating electric currents resulting in the generation of a strain-specific genomic fingerprint (Cantor et al. 1988; Mathew et al. 1988; Tanskanen et al. 1990). PFGE has been used extensively to characterise members of both *L. lactis* and *S. thermophilus* (Boutrou et al. 1995; Ward et al. 2004; Kelly et al. 2010; Zinno et al. 2010). PFGE is highly discriminatory in comparison to other methods, but the procedure is time-consuming, technically challenging, and the lack of fingerprint databases limits its widespread use in the dairy industry. Similar to PFGE, AFLP involves the digestion of chromosomal DNA with restriction endonucleases. Following digestion, oligonucleotide adapters containing a core sequence and a sequence homologous to the sticky ends produced by the endonuclease are added to the DNA fragments creating a target site for the AFLP-specific primers. These primers are designed to match the core sequence and the restriction enzyme-specific sequence (Vos et al. 1995; Vuylsteke et al. 2007). Two to three additional non-specific bases may be added to the 3′-end of the primer to increase the selectivity of the protocol. The AFLP method can be automated by the use of fluorophore-labelled primers, coupled to fragment separation by denaturing gel electrophoresis and subsequent detection of AFLP fragments using an automated sequencer (Gancheva et al. 1999). While initially labour-intensive, the development of a particular protocol can be used to characterise hundreds of bacterial strains using only small amounts of template DNA as well as allowing for faster processing of samples and a high-throughput analysis of strains without any prior knowledge of the genome (Paun and Schönswetter 2012). However, such approaches require an automated DNA sequencer which can be expensive to purchase, run and maintain. To date, there has been no reported use of AFLP in characterising *L. lactis* strains used as dairy starter cultures. Analysis of dairy *S. thermophilus* strains using AFLP indicated that this approach provides a higher degree of differentiation at both the species and strain level compared to other methods such as RAPD PCR (Lazzi et al. 2009).

### PCR-based methods

RAPD PCR is a fingerprinting technique which uses ten nucleotide (10-mer) primers to amplify random segments of the bacterial genome without the requirement of having previous sequence knowledge of the genome (Williams et al. 1990). Diversification or discrimination at the intraspecies and interspecies level as well as the identification of mutations within a genome is based on whether or not the primer will amplify a segment of DNA resulting in the generation of unique DNA banding patterns. The use of RAPD PCR has been extensively applied to the characterisation of members of the LAB, as well as lactococcal and streptococcal phages (Rossetti and Giraffa 2005; Rodríguez et al. 2008; Chouayekh et al. 2009). RAPD PCR has been shown to differentiate the lactococcal sub-species and also members of the same subspecies, particularly where members of a particular subspecies display different phenotypic characteristics (Tailliez et al. 1998; Samarzija et al. 2002). PCR-RFLP, which utilises a combination of PCR and a REase-based approach, is a genotyping technique that relies on the presence of single nucleotide polymorphisms (SNPs) or multi-nucleotide polymorphisms (MNPs) within a specific region of targeted homologous DNA (Saiki et al. 1985). The presence of SNPs or MNPs within the amplified region of interest can alter the recognition site of the REase resulting in different banding patterns, allowing the discrimination of different (sub)species within a bacterial genus/species, or members of the same species. PCR-RFLP has been employed to discriminate between *L. lactis* ssp. *lactis* and *L. lactis* ssp. *cremoris* by targeting different genes such as those specifying N-acetylmuramidase (*acmA*), the 16S rRNA and glutamate decarboxylase (*gadB*) (Buist et al. 1995; Ward et al. 1998; Nomura et al. 2002). Recently, two additional gene targets, the genes encoding a serine protease (*htrA*) and a non-proteolytic protein peptidase family M16 member (*yueF*) were found to be more suitable for differentiating between the two subspecies *cremoris* and *lactis* when used in combination with the REases TaqI and AluI, respectively (Khemariya et al. 2013). Both RAPD and PCR-RFLP target specific DNA regions, whereas rep-PCR employs sets of primers that target non-coding repetitive short polynucleotide sequence tracts that can be found scattered across the bacterial genome (Stern et al. 1984; Versalovic et al. 1991). Amplification of these repeating sequences results in the generation of fragments of various lengths depending on the length of the DNA located between these repetitive elements generating strain-specific patterns when separated by gel electrophoresis. Several short repetitive elements can be used as targets for amplification including the repetitive extragenic palindromic (REP) elements; enterobacterial repetitive intergenic consensus (ERIC) sequences (127-bp imperfect palindromes) and BOX elements (modular DNA segments encompassing three subunits, A-C, which can exist in various combinations) (Koeuth et al. 1995; Tobes and Ramos 2005; Wilson and Sharp 2006). While it has been demonstrated that rep-PCR is comparable to RFLP and RAPD, several limitations such as poor resolution and band separation have been observed for rep-PCR (Olive and Bean 1999). To overcome these limitations, fluorophore-labelled oligonucleotides can be used where the amplified sequences are separated using a DNA sequencer similar to the AFLP approach (Versalovic et al. 1995), and this approach has been used to characterise *S. thermophilus* strains and establish links between *S. thermophilus* strains and their geographical origin (Brusetti et al. 2008). There have been mixed reports on the differentiating ability of rep-PCR. While Urbach et al. (1998) applied rep-PCR for the characterisation of *L. lactis* strains and reported it as a useful and reproducible technique at differentiating strains, subsequent studies have shown that rep-PCR cannot distinguish between ssp. *lactis* and ssp. *cremoris* (Prodělalová et al. 2005).

### Multilocus sequencing typing (MLST)

MLST is a technique that targets the internal region (400–500 bp) of several housekeeping genes and is now by many considered to represent the gold standard for species differentiation (Maiden et al. 1998). MLST has been used to characterise both *L. lactis* and *S. thermophilus* strains, including wild-type and industrial starters. MLST has been shown to effectively discriminate collections of lactococcal strains into subgroups or clusters, as well as differentiating the sub-species based on housekeeping genes such as peptidase N and X-prolyl dipeptidyl aminopeptidase (Fernández et al. 2011). MLST can be used to separate lineages among the sub-species of *L. lactis*, although some lineages were found to include representatives of both subsp. *lactis* and *cremoris*, suggesting that MLST is not completely discriminatory at the sub-species level (Rademaker et al. 2007). In a recent study, the MLST scheme for *L. lactis* was revised based on additional genes that were not necessarily housekeeping genes, particularly genes that act as indicators of species divergence (Passerini et al. 2010). MLST has also been applied to differentiate isolates of *S. thermophilus* using primers that target eight housekeeping genes, previously applied for MLST purposes on strains of *Streptococcus salivarius* (Delorme et al. 2007, 2010).

### Cell wall polysaccharide (CWPS) operon typing

The (Gram-positive) lactic acid bacterial cell envelope represents a complex structure composed of a thick peptidoglycan layer, teichoic acids, cell wall polysaccharides (CWPS) and various surface carbohydrates (Chapot-Chartier 2014). Recently, it has been demonstrated by a multiplex PCR approach that, based on the genetic composition of their CWPS-encoding gene cluster, (most) lactococcal dairy strains can be assigned to one of three types (types A, B and C) (Mahony et al. 2013). Strains possessing the CWPS C type can be further divided into five subtypes (designated C_1_ through to C_5_), which can also be identified by multiplex PCR (Ainsworth et al. 2014b). These PCR-based approaches are important and rapid in classifying the CWPS biosynthesis cluster, in particular from phage sensitivity prediction and strain blend derivation perspectives.

## Next-generation sequencing (NGS) approaches

Sanger sequencing was first described in the 1970s by Frederick Sanger and colleagues (Sanger et al. 1977) and became the dominant method of sequencing DNA for the remainder of the 20th century. It was used to sequence the complete human genome (Lander et al. 2001; Venter et al. 2001) and is a highly accurate method of sequencing. However, Sanger sequencing, referred to as a “first generation” sequencing method, is expensive and impractical for large sequencing projects, and in recent times, this method has partly been replaced by “next-generation” sequencing (NGS) methods (Metzker 2010).

NGS methods are high-throughput DNA sequencing technologies permitting the sequencing of millions of DNA strands in parallel generating large volumes of sequence data in a relatively short period of time (Pettersson et al. 2009). There are currently a number of methods in use (Table 1), but for the purposes of this review, we will briefly compare four of the most commercially viable methods, namely Roche 454-pyrosequencing, Illumina-Solexa, Life Sciences Ion-Torrent and Pacific Biosciences Single-molecule real-time sequencing (SMRT). For more in-depth information on each of these technologies, see Loman et al. (2012a, b).

**Table 1:** Comparison of next-generation sequencing technologies adapted from Metzker (2010)

Platform	Library preparation	Chemistry	Consensus accuracy	Average read length (bp)	Reads per run	Run time	Pros	Cons	Application	References
Roche 454 GS FLX Titanium+	Fragment, Mate Pair/ emPCR	Pyrosequencing	99.997%	~700, maximum 1000	~1,000,000	23 h	Long read length	High reagent cost, homopolymer repeat errors	De novo assemblies, metagenomics	http://454.com/products/gs-flx-system/index.asp
Illumina- Solexa MiSeq	Fragment, Mate Pair/ Solid phase	Reversible terminator	98%	2 × 300	~25,000,000	~55 h 300 bp reads	Widely used platform	Short read length	Small genomes, 16s amplicon, improving coverage	http://systems.illumina.com/systems/miseq.html
Life Technologies SOLiD 5500 Series	Fragment, Mate Pair/ emPCR	Sequencing by ligation (Cleavable probe)	99.99%	Mate-paired 2 × 60, Paired-end 75 × 35 Fragment 75	1.2–1.4 billion	1–2 weeks	Low-cost	Slow, issues with palindromic sequences reported	Whole genome re-sequencing, variant analysis	http://www.lifetechnologies.com/ (Huang et al. 2012)
Life Technologies Ion Torrent	Fragment, Mate Pair/ emPCR	Sequential ion detection	98%	35–400	80,000,000	90 min	Fast and inexpensive	Reported homopolymer errors	Small genomes, gene expression, ChiP-SEQ	http://www.lifetechnologies.com/
Pacific Biosciences SMRT *RS* II	Fragment only/ Single molecule	Real-time	99.999%	N50 14,000, maximum >40,000	~50,000	30 min–4 h	Longest read length, detects base modifications, fast	Low single sequence accuracy 87%	De novo assemblies, Base modification detection, Transcriptome sequencing	(Murray et al. 2012; Chin et al. 2013) http://www.pacificbiosciences.com/

### Comparison of NGS approaches

There are a number of next-generation techniques available with associated advantages and disadvantages to each technique depending on the desired application (Table 1). For the study of lactococcal or streptococcal starter cultures, any of the aforementioned techniques may be applied to obtain finished genome sequences due to the small genome size of these species: strains of *L. lactis* typically possess a ~2.5 Mb chromosome, whereas *S. thermophilus* strains have a smaller genome of around 1.8 Mb.

454-Pyrosequencing is a next-generation, high-throughput sequencing methodology based on the sequence by synthesis approach and is useful due to its longer read length compared to read lengths generated by the current Illumina or Ion-torrent platforms: 700 bp compared to 300 and 400 bp, respectively. While 454-pyrosequncing has been used extensively over the last 10 years, it was announced in 2013 that Roche will phase out this sequencing platform by mid-2016.The reasons for this discontinuation of 454-pyrosequencing include the advent of lower cost, high(er) throughput sequencing technologies, along with increasing read lengths of the alternative NGS technologies (Chaisson et al. 2009). Errors in homopolymer sequence tracts have also been reported with the 454-pyrosequencing method (Gilles et al. 2011) and ion torrent technology (Loman et al. 2012b).

The Ion-Torrent PGM represents a low-cost and rapid sequencing methodology generating around 80 million sequence reads in a single run of approximately 90 min. The Illumina approach has been one of the most widely used sequencing approaches in recent years and can generate a large volume of sequencing data (Metzker 2010), although the average read length is relatively low, in particular when compared to the newer PacBio SMRT platform. Current Illumina sequencing-by-synthesis (SBS) instruments are capable of generating over 1 terabase of data in a single run and are capable of sequencing bacterial genomes in a matter of hours. These properties, combined with low sequencing costs, have in recent years made Illumina become the dominant sequencing technology.

The PacBio SMRT approach has the advantage of the longest read lengths of any sequencing technology currently in use (Chin et al. 2013), with Pacific Biosciences reporting N50 read lengths of >14,000 bp and maximum read lengths of >40,000 bp, which is extremely useful for covering repetitive regions of genomes, particularly so in lactococcal genomes where a large number of insertion sequence (IS) elements cause problems during sequence read assembly (Daveran-Mingot et al. 1998; Chopin et al. 2001; Kok et al. 2005). The SMRT sequencing approach also moves beyond traditional detection of the four DNA bases as it is the first high-throughput approach to directly detect DNA base modifications (Flusberg et al. 2010). This allows SMRT sequencing to differentiate between unmodified bases and those with m6A, m4C or m5C base modifications (Clark et al. 2012). One drawback of the PacBio SMRT platform which should be considered is the reports of higher error rates compared to other NGS platforms. Since launching the SMRT platform, Pacific Biosciences have addressed this issue by incorporating circular consensus sequencing (CCS), which has led to greatly reduced error rates (Hodkinson and Grice 2015) and achieving a higher consensus accuracy, currently reported at 99.999% by Pacific Biosciences. In comparison to early studies which reported a sequencing inaccuracy rate of ~13–18% (Quail et al. 2012; Mosher et al. 2013), more recent studies have reported a large reduction in these rates (Schloss et al. 2015).

As discussed, the PacBio SMRT platform currently possesses a number of unique advantages over other NGS methods. However, a noteworthy new single molecule sequencing method currently in development is nanopore sequencing which may challenge PacBio’s dominance in this area. Nanopore sequencing is predicted to deliver long read lengths and base modification data, while the simple sample preparation and possibility of label-free DNA sequencing are expected to reduce sequencing costs dramatically (Clarke et al. 2009).

Genotypes of lactococcal and streptococcal strains derived from genomics (Table 2) can provide a myriad of information about industrially important traits. There is an impressive array of tools available for post-sequencing and comparative genome analyses, and readers requiring more information should refer to Edwards and Holt (2013). Here, we discuss some of the key genetic markers derived from genomic analysis which can be used for strain selection with particular emphasis on phage resistance and flavour development.

**Table 2:** Sequencing methods used for finished quality lactococcal and streptococcal genomes available from the NCBI (correct January 2014)

Organism	GenBank accession	Sequencing technology	Year	Reference
*L. lactis*				
ssp. *lactis* IL1403	GCA_000006865.1	Sanger	2001	Bolotin et al. 2001
ssp*. cremoris* SK11	GCA_000014545.1	Sanger	2006	Makarova et al. 2006
ssp. *cremoris* MG1363	GCA_000009425.1	Sanger	2007	Wegmann et al. 2007
ssp. *lactis* KF147	GCA_000025045.1	Combined 454-pyrosequencing & Illumina	2009	Siezen et al. 2010
ssp. *cremoris* NZ9000	GCA_000143205.1	Illumina	2010	Linares et al. 2010
ssp. *cremoris* A76	GCA_000236475.1	Sanger	2011	Bolotin et al. 2012
ssp. *lactis* CV56	GCA_000192705.1	454-pyrosequencing	2011	Gao et al. 2011
ssp. *lactis* IO-1	GCA_000344575.1	Sanger	2012	Kato et al. 2012
ssp*. cremoris* UC509.9	GCA_000312685.1	Combined 454-pyrosequencing & Illumina	2012	Ainsworth et al. 2013
ssp. *cremoris* KW2	GCA_000468955.1	454-pyrosequencing	2013	Kelly et al. 2013
ssp. *lactis* KLDS 4.0325	GCA_000479375.1	Illumina	2013	Yang et al. 2013
ssp*. lactis* NCDO 2118	GCA_000478255.2	SOLiD, Ion PGM & Ion Torrent PGM	2014	Oliveira et al. 2014
ssp. *lactis* SO	GCA_000807375.1	Ion Torrent PGM	2014	McCulloch et al. 2014
ssp. *lactis* AI06	GCA_000761115.1	454-pyrosequencing	2014	Unpublished
*S. thermophilus*				
CNRZ1066	GCA_000011845.1	Sanger	2004	Bolotin et al. 2004
LMG 18311	GCA_000011825.1	Sanger	2004	Bolotin et al. 2004
LMD-9	GCA_000014485.1	Sanger	2006	Makarova et al. 2006
ND03	GCA_000182875.1	Combined 454-pyrosequencing & Illumina	2010	Sun et al. 2011
JIM 8232	GCA_000253395.1	Sanger & SOLiD	2011	Delorme et al. 2011
MN-ZLW-002	GCA_000262675.1	Combined 454-pyrosequencing & Illumina	2012	Kang et al. 2012
ASCC 1275	GCA_000698885.1	454-pyrosequencing	2014	Wu et al. 2014

### Practical implications of genomics-driven characterisation of dairy cultures

#### Metabolic capabilities

Dairy LAB encode a number of key metabolic pathways that are necessary for the production of cheese and other fermented dairy products. These include genes required for lactose utilisation, degradation of peptides and amino acids, citrate metabolism and lipolysis, all of which can be characterised at a genetic level given the availability of sequencing data.

#### Lactose and lactate metabolism

The gene products of the *lac* operon facilitate and govern lactose utilisation in LAB and provide dairy strains with the ability to rapidly ferment lactose required for growth in milk. In *L. lactis*, the plasmid-bourne *lac* operon consists of the genes *lacABCDEFGX* and is regulated by a repressor, encoded by the adjacent *lacR* gene (van Rooijen and de Vos 1990; van Rooijen et al. 1992). Loss of the *lac* operon has been reported due to the instability of the large extra-chromosomal element on which it is encoded (McKay et al. 1972; Ainsworth et al. 2014c), resulting in spontaneous mutants that are incapable of growth in milk. Interestingly, the plasmid-cured laboratory strain *L. lactis* MG1363, which does not harbour the *lac* operon, is capable of growth on lactose-supplemented media following prolonged adaptation due to the activity of a cellobiose-specific phosphotransferase system (PTS), which can act as an alternative lactose utilisation pathway (Solopova et al. 2012). Another example of an alternative lactose metabolic pathway is found in the slow lactose fermenter *L. lactis* NCDO2054 which metabolises lactose via the Leloir pathway (Bissett and Anderson 1974). This occurs as a result of *lacA*, which encodes a galactoside acetyltransferase, and *lacZ*, which encodes a β-galactosidase, being integrated into the *gal* (galactose) operon (Vaughan et al. 1998). Such data suggests that phenotypic growth on lactose may not be an absolutely reliable indicator for the presence of the *lac* operon within lactococcal strains. Further studies have suggested that certain PCR-based techniques may also be somewhat unreliable in indicating the true lactose genotype.

A recent study by Ferrario et al. (2012) reported on the screening for isolates of *L. garvieae* in the dairy environment using primers targeting the *lacG* gene. They found that *lacG* is variably present among *L. garvieae* isolates from the meat environment and is not limited to dairy isolates, demonstrating the need for complete genome sequences for the correct identification of dairy isolates.

Lactose metabolism by *S. thermophilus* differs from that observed in *Lactococcus* ssp.: lactose is transported into the cell by the secondary transport system LacS in antiport with galactose (Delcour et al. 2000). The *lacS* gene is organised in an operon with the β-galactosidase-encoding gene, *lacZ*, similar to the system in *Lactobacillus delbrueckii* (Schroeder et al. 1991). The *lacZ* gene in *S. thermophilus* is well conserved and has been used for PCR-based identification purposes of *S. thermophilus* from fermented dairy products (Lick et al. 1996) and for the isolation of folate-producing *S. thermophilus* strains from Indian fermented milk products (Iyer et al. 2011).

#### Citrate metabolism

Citrate metabolism in dairy fermentations conducted by citrate-positive (Cit^+^) lactococci and *Leuconostoc* spp. is important as it leads to the production of a number of volatile flavour compounds (McSweeney and Sousa 2000). Citrate uptake and subsequent diacetyl production are governed by the plasmid-encoded *citQRP* operon in lactococcal species (Drider et al. 2004). It has been demonstrated that the *citP* gene is well conserved amongst LAB with approximately 98% amino acid identity making it a useful screening target for Cit^+^ starters (Drider et al. 2004). Lactococci capable of metabolising citrate are classified as *L. lactis* subsp. *lactis* biovar diacetylactis (Kelly et al. 2010), a classification that has led to confusion since plasmid-encoded characteristics such as citrate and arginine metabolism can be transferred to subsp. *cremoris* strains leading to incorrect characterisation based on phenotype (Kelly et al. 2010). It is also noteworthy that recent studies have indicated potential adverse health effects associated with diacetyl production, which may lead to the future removal of diacetyl-producing LAB from starter cultures (Shibamoto 2014).

#### Proteolysis

Proteolysis and the degradation of casein from milk are one of the most important contributors to flavour development in cheese (McSweeney 2004). Lactococcal strains contribute to proteolysis through the hydrolysis of casein by peptidases and proteases and the catabolism of peptides and amino acids from casein breakdown (Steele et al. 2013). There are a number of genes which contribute to this function, such as various and mostly chromosomally specified peptidase-encoding genes (e.g. *pepC*, *pepN*, *pepX*, *pepP*, *pepA*, *pepF2*, *pepDA1*, *pepDA2*, *pepQ*, *pepT*, *pepM* and *pepO1*), the plasmid-encoded *opp* operon, which specifies an oligopeptide-uptake system, and the plasmid-bourne gene that specifies the *L. lactis* cell wall-associated protease PrtP, required for the proteolytic phenotype (Yu et al. 1996). The majority of the genes mentioned above are monocistronic (e.g. *pepC*, *pepN* and *prtP*) or co-transcribed, such as *opp* and *pepO1*, while *pepF2*, *pepM* and *pepT* are transcribed with genes that are (apparently) unrelated to proteolysis (Guédon et al. 2001). There are also a number of uncharacterised proteins which contain peptidase-associated domains, many of which are strain-specific and their roles may become clearer as more genome sequences become available (Siezen et al. 2005).

As discussed, proteolysis contributes greatly to cheese flavour development; however, high levels of proteolysis can also cause bitterness in cheese (Broadbent et al. 2002). The *L. lactis* extracellular cell wall proteinase (lactocepin) has been shown to be directly involved in the bitter flavour defect in cheddar cheese varieties, specifically starters which produce lactocepin of the so-called group a, e or h (Broadbent et al. 2002). Broadbent et al. (2002) concluded that the bitterness defect in cheese could be altered through gene exchange or replacement in the starter culture. These findings highlight the benefits of subsp. *cremoris* strains in lactococcal starter cultures in comparison to subsp. *lactis*.

A recent study by Liu et al. (2010) indicates that our knowledge of the proteolytic system in LAB can be enhanced by systematic genome-wide studies of the regions encoding proteins involved in proteolysis. These authors indicated that comparative genomics can be used to distinguish various sub-groups within protein superfamilies involved in proteolysis where the generated information predicts the proteolytic ability of LAB strains. A major finding from this study was the confirmation of proteolytic diversity among ssp. *lactis* and ssp. *cremoris* strains and the provision of a genetic basis for this diversity, linked to distinct patterns in the presence or absence of genes encoding proteolytic functions (Liu et al. 2010).

#### Lipolysis

Lipolysis involves the breakdown of milk fats and hydrolysis of triglycerides into lipids and fatty acids, activities that are considered to be crucial for flavour development in cheese production, particularly in the production of cheddar varieties (McSweeney and Sousa 2000). In LAB, lipolytic enzymes involved in lipolysis are mainly esterases and lipases belonging to a class of enzymes called the carboxylic ester hydrolases (Verger 1997). Apparently, *estA* is the only esterase-encoding gene in *L. lactis*, being capable of hydrolysing short chain fatty acid esters (Nardi et al. 2002). However, this research area of cheese flavour development remains considerably under-represented in lactococcal studies compared to those related to proteolysis (McSweeney and Sousa 2000).

Therefore, a genomics approach may be beneficial in broadening our scope of knowledge on lipolysis in lactococcal and streptococcal strains as demonstrated in other LAB.

### Phage robustness

#### Prophages

As mentioned previously, it is possible to determine the presence of prophages by phage induction assays (Chopin et al. 2001). However, such approaches are time-consuming and require the assessment of large collections of strains. In addition, ‘true’ prophage induction can only be determined using additional methods such as confirmation of the presence of prophages by performing phage sensitivity assays upon identification of a sensitive host strain, PCR or flow cytometry (Sozhamannan et al. 2006). Whole genome sequencing can readily identify the presence of temperate phages within the host genome. Furthermore, the availability of programmes such as Phage_Finder as well as gene annotation tools aid in the determination of the presence of intact or cryptic prophage elements (Fouts 2006). The presence of prophages is more common in *L. lactis* than *S. thermophilus* with some of the former harbouring six prophages (Chopin et al. 2001; Wegmann et al. 2007). Lysogenic *S. thermophilus* strains have been identified and have been correlated with strain autolysis (Husson-Kao et al. 2000; Neve et al. 2003). While the presence of prophages in commercial strains has generally been considered an undesirable trait due to the risk of phage excision, some prophage elements have been found to encode systems which are beneficial for phage defence namely, superinfection exclusion systems (Sie) in lactococcal strains and Lipoprotein (Ltp) in streptococcal strains (Gasson and Davies 1980; McGrath et al. 2002; Mahony et al. 2008).

#### Restriction-modification (R-M) systems and abortive infection (Abi) systems

Genes encoding R-M systems are present on approximately 90% of currently available bacterial and archaeal genome sequences (Roberts et al. 2003). These systems can be plasmid or chromosomally encoded, and their general role is to recognize and target invading foreign DNA with restriction enzymes, while simultaneously protecting the host’s DNA by methyltransferase (MTase) activity. Four types of R-M systems (I, II, III and IV) are currently recognized and have been extensively reviewed previously (Roberts et al. 2003; Loenen et al. 2013; Pingoud et al. 2014; Rao et al. 2014). The presence of various R-Ms in industrial starter cultures plays an important role in phage defence, as invading phage DNA, if unmethylated (except in the case of type IV R-M systems), will be subject to endonuclease activity. Traditional and laborious approaches to the identification of R-Ms such as the use of crude cell extracts, extract fractionation and restriction endonuclease assays are time-consuming and often only suitable for type II R-Ms such as the lactococcal ScrFI, LlaBAI and LlaBI (Fitzgerald et al. 1982; Nyengaard et al. 1993; Mruk et al. 2003) and *S. thermophilus* Sth4551 (Guimont et al. 1993). These methods have been replaced with the emergence of more accessible sequencing technologies allowing for the prediction of chromosomal- and plasmid-encoded R-Ms through similarity searches, e.g. the *L. lactis* systems LlaJI, LldI and LlaI (Hill et al. 1989; Deng et al. 2000; O'driscoll et al. 2004) and the *S. thermophilus* R-M system Sth3681 (Burrus et al. 2001). In recent years, the emergence of SMRT sequencing technology (as discussed above) has revolutionised the identification of whole genome modification and the function of R-Ms. Combining whole genome sequencing and MTase motif analysis, the functions of one or more bacterially encoded R-Ms can be predicted which can then be confirmed using heterologous gene expression coupled with restriction endonuclease assays. This approach has been applied to both bacteria and bacteriophages alike (Murphy et al. 2014; O'Connell-Motherway et al. 2014), though it has not yet been applied to lactococcal or streptococcal strains.

Abortive infection (Abi) systems are host-encoded resistance mechanisms that disrupt critical stages in the lytic phage cycle such as phage transcription, translation, DNA replication or phage DNA packaging and have been extensively studied in *L. lactis* (Ainsworth et al. 2014a, c). Abi-mediated resistance typically culminates in the death of the infected host cell in order to limit the release of progeny particles, thus protecting the neighbouring bacterial population. Currently, 23 Abi systems (AbiA-AbiZ) are known for *L. lactis*, which, with the exception of AbiN and AbiV, are all plasmid-encoded (Prevots and Ritzenthaler 1998; Chopin et al. 2005; Ainsworth et al. 2014a, c). The presence of Abi systems was first identified due to the protective effect that certain lactococcal plasmids have against phage infection, by causing a decreased burst size and an altered phage plaque morphology as observed for plasmids pTR2030 and pIL105 (Sing and Klaenhammer 1986; Gautier and Chopin 1987). Subsequently, plasmids that conferred such resistance to infecting phages were digested with restriction endonucleases and the fragments cloned into suitable shuttle vectors. The various recombinant derivatives were then screened to determine if a particular fragment provided phage resistance as observed for AbiE and AbiF encoded on the lactococcal plasmid pNP40 (Garvey et al. 1995). Such experimental approaches are time-consuming, particularly if the approach was to be employed in order to assess a collection of dairy starters. As with R-Ms, whole genome sequencing can readily identify both chromosomally and plasmid-encoded Abi systems, in particular in the case of SMRT sequencing technology, which in many cases can produce completed, single contig bacterial genome and plasmid sequence information for use in comparative sequence analyses (Sistla and Rao 2004).

#### CRISPR/Cas systems

Clustered regularly interspaced short palindromic repeats (CRISPR) and CRISPR-associated (Cas) genes form an acquired adaptive immunity system against foreign genetic elements in prokaryotes (Horvath et al. 2008; van der Oost et al. 2009; Deveau et al. 2010). CRISPR systems are composed of a series of conserved repeats which are separated by protospacers, variable sequences involved in target recognition, an A-T-rich leader region located at the 5′ end of the CRISPR locus and Cas genes (Deveau et al. 2008). CRISPR systems play an important role in phage resistance in dairy starter strains (Mills et al. 2010), and furthermore, CRISPR systems can be used as a tool for the typing and comparative analyses of strains of *S. thermophilus* (Horvath et al. 2008). CRISPR typing of *S. thermophilus* performed by Horvath et al. (2008), based on a combination of primers targeting conserved regions and Sanger sequencing resulted in the identification of CRISPR3 and demonstrated the diversity of CRISPR systems across 124 *S. thermophilus* strains. To date, there have been four distinct CRISPR loci identified in *S. thermophilus*, designated as CRISPR1 through to CRISPR4 (Makarova et al. 2011). In *L. lactis*, only one plasmid-encoded CRISPR/Cas locus has been characterised (Millen et al. 2012).

## Future perspectives and conclusions

While it is likely that “omics”-based technologies will never completely replace traditional culture-based methods, there is a vast array of knowledge to be gained from integrating these disciplines. Small-scale trial fermentations will continue to be the only genuine test of the performance of starter cultures within an industrial setting; yet, it is an impractical approach for screening large culture banks for suitable strains. The recent advances in NGS technologies have ensured that sequencing has become a more accessible avenue that may permit a rational approach to reduce the number of potential candidates for such trials, and to minimize screening times and labour-intensive culture techniques. Further combinations of sequencing-based approaches with other “omics”-based technologies, such as transcriptomics and proteomics in cheese, may help to moderate the genotype-phenotype link in the future.

Genome decay and redundancy, as highlighted in dairy lactococcal isolates (Makarova et al. 2006; Goh et al. 2011; Ainsworth et al. 2013), coupled to *cremoris* type strains which are believed to be descended from a few closely related lineages (Kelly et al. 2010), are factors likely to limit the selection of novel starter strains in the future. This is perpetuated by the likelihood of large redundancies in culture collections from different institutes globally and the differentiation of many of these strains. Additionally, the possibility of incorrect phenotype/genotype association, such as the plasmid-encoded citrate metabolism trait, is likely to only be resolved by complete genome sequencing.
